# Perineural invasion in colorectal cancer: mechanisms of action and clinical relevance

**DOI:** 10.1007/s13402-023-00857-y

**Published:** 2023-08-23

**Authors:** Hao Wang, Ruixue Huo, Kexin He, Li Cheng, Shan Zhang, Minhao Yu, Wei Zhao, Hui Li, Junli Xue

**Affiliations:** 1 Department of Oncology, Shanghai East Hospital, School of Medicine, Tongji University, Shanghai, 200092 P.R. China; 2 State Key Laboratory of Oncogenes and Related Genes, Ren Ji Hospital, School of Medicine, Shanghai Cancer Institute, Shanghai Jiao Tong University, Shanghai, 200240 P.R. China; 3 Department of Gastrointestinal Surgery, Ren Ji Hospital, School of Medicine, Shanghai Jiao Tong University, Shanghai, 200217 P.R. China

**Keywords:** Perineural invasion, Prognosis, Tumor microenvironment, Neurotrophins, Colorectal innervation

## Abstract

**Background:**

In recent years, the significance of the nervous system in the tumor microenvironment has gained increasing attention. The bidirectional communication between nerves and cancer cells plays a critical role in tumor initiation and progression. Perineural invasion (PNI) occurs when tumor cells invade the nerve sheath and/or encircle more than 33% of the nerve circumference. PNI is a common feature in various malignancies and is associated with tumor invasion, metastasis, cancer-related pain, and unfavorable clinical outcomes. The colon and rectum are highly innervated organs, and accumulating studies support PNI as a histopathologic feature of colorectal cancer (CRC). Therefore, it is essential to investigate the role of nerves in CRC and comprehend the mechanisms of PNI to impede tumor progression and improve patient survival.

**Conclusion:**

This review elucidates the clinical significance of PNI, summarizes the underlying cellular and molecular mechanisms, introduces various experimental models suitable for studying PNI, and discusses the therapeutic potential of targeting this phenomenon. By delving into the intricate interactions between nerves and tumor cells, we hope this review can provide valuable insights for the future development of CRC treatments.

## Introduction

Colorectal cancer (CRC) consistently ranks as one of the most prevalent malignant gastrointestinal tumors and remains a leading cause of cancer-related deaths [1]. Despite advances in surgical techniques and adjuvant chemoradiotherapy, the long-term survival rates for CRC patients remain unsatisfactory [2, 3]. According to data published in the Journal of the American Medical Association, approximately 20% of CRC patients present with metastases at the time of diagnosis, and 25% of patients with focal lesions will develop metastases later [4]. Tumor metastasis significantly contributes to a poor prognosis, with less than 20% of patients with metastatic CRC surviving beyond five years. Therefore, it is crucial to comprehend the mechanisms underlying tumor metastasis and develop effective strategies to prevent or treat it.

Tumor invasion and metastasis depend on various components in the tumor microenvironment (TME) [5, 6]. While the involvement of blood vessels and lymphatic vessels in tumor growth and invasion is well-established, the role of nerves has been largely underestimated. Accumulating evidence suggests that the activation of neural growth within tumors, known as neoneurogenesis, is another key driver of cancer progression [7]. Several studies have demonstrated that the crosstalk between the tumor and nerves synergistically promotes tumor development [8–10]. On the one hand, neurons provide neurotransmitters, angiogenic signals, immunogenic compounds, and growth factors that can be utilized by tumor cells, creating a microenvironment conducive to the survival and proliferation of tumor cells. Simultaneously, nerves serve as “channels” for tumor dissemination and metastasis. On the other hand, tumor cells can also secrete neurotrophic factors and axon guidance factors, promoting the internal innervation process of the tumor.

Perineural invasion (PNI) is one of the most potent interactions between tumors and nerves. According to Batsakis (1985), PNI is defined as “tumoral invasion in, around, and through the nerves” [11]. While this definition is widely accepted, it does have certain limitations. An alternative and broader definition, advocated by Liebig et al., includes tumors in close proximity to nerves involving at least 33% of the nerve circumference or the presence of tumor cells within the epineurium, perineurium and endoneurium of the nerve sheath [12]. Anything less than 33% represents focal abutment and not invasion [13]. PNI represents the pinnacle of tumor-nerve interaction, providing both tumor cells and nerves with a survival advantage.

PNI is a relatively novel histopathological feature associated with poor clinical outcomes and decreased survival in various malignancies, including pancreatic ductal adenocarcinoma (PDAC), head and neck squamous cell carcinoma (HNSCC), prostate cancer, gastric cancer, and CRC [9, 12, 14]. In most studies, the reported incidence of PNI in CRC patients ranges between 9% and 33% [15–18], which is significantly lower than that observed in PDAC. However, once confirmed, PNI is linked to rapid disease progression and unfavorable outcomes [19]. The New England Journal of Medicine has recognized PNI as pathological evidence of early metastasis, which is independently associated with decreased survival in CRC [20].

The interactions involving neural effects on other cells in the TME have garnered increasing attention [21]. Recently, an insightful perspective article discussed the relationship between the nervous system and the development of hallmark capabilities in tumors, emphasizing the role of neurons and axons as constituents of the TME that can facilitate the acquisition of hallmark features by tumors [22]. However, a comprehensive understanding of the interaction between PNI and the TME in CRC remains lacking. In this review, our aim is to elucidate the clinical relevance of PNI and provide in-depth overview of the specific molecules and cells within the TME that regulate PNI and promote cancer development. We hope that this endeavor will contribute to a deeper comprehension of the significance and underlying mechanisms of PNI in CRC.

## Colorectal innervation

**Fig. 1:**
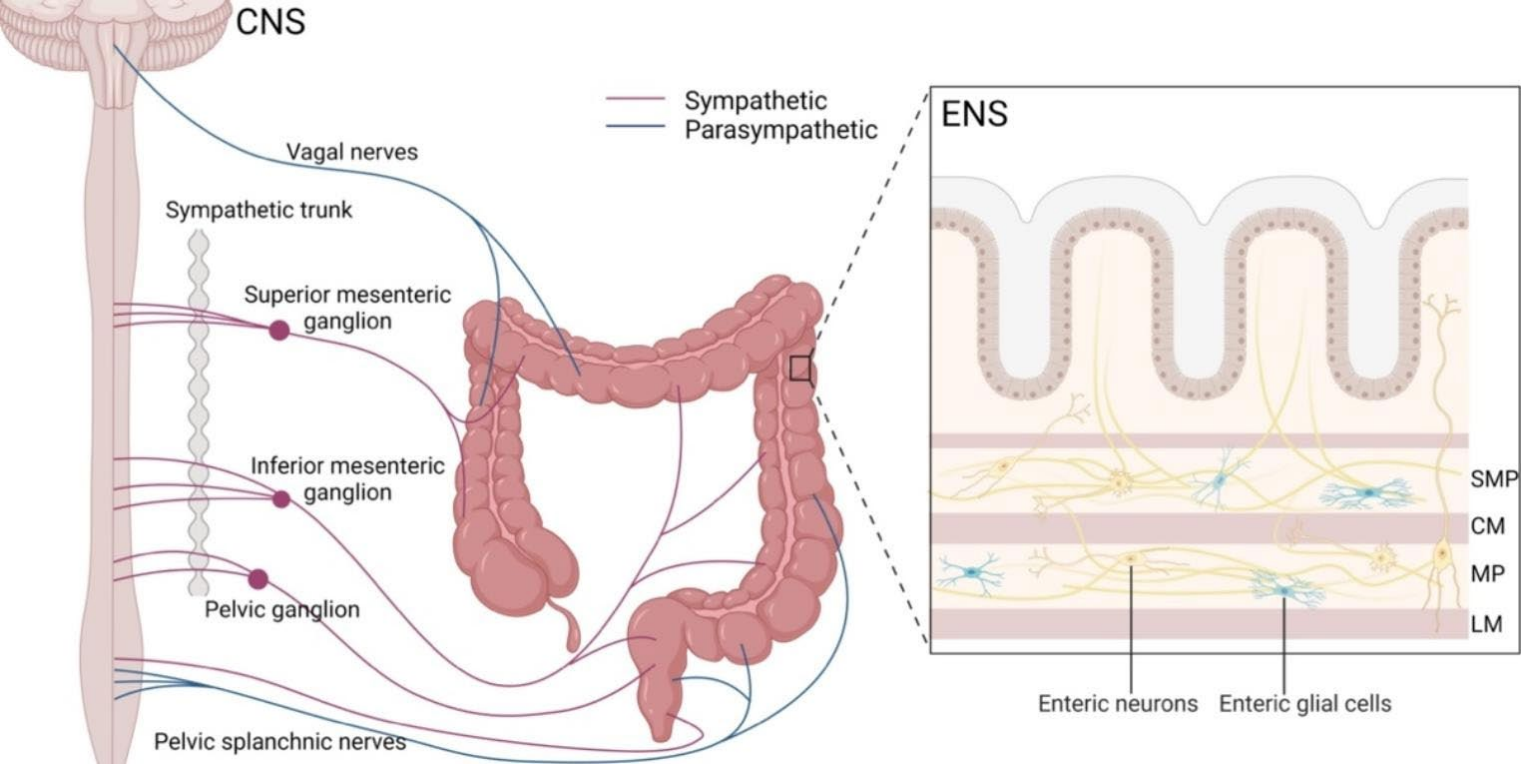
Extrinsic and intrinsic innervation of the colorectal tract. Extrinsic innervation incorporates sympathetic and parasympathetic/vagal input from the CNS. Intrinsic innervation is provided by the ENS, which innervates the entire gut wall via enteric neurons and enteric glial cells. Abbreviations: CNS, central nervous system; ENS, enteric nervous system; SMP, submucosal plexus; CM, circular muscle; MP, myenteric plexus; LM, longitudinal muscle

The colon and rectum are highly innervated organs, with nerve distribution categorized into two groups: extrinsic innervation and intrinsic innervation (Fig. 1).

### Extrinsic innervation

Extrinsic innervation refers to the autonomic innervation of the gut, where neuronal cell bodies are located outside the gut and incorporate sympathetic and parasympathetic/vagal input from the central nervous system (CNS). Sympathetic fibers have their cell bodies located in the sympathetic chain and in prevertebral ganglia next to the spinal cord. They typically travel along blood vessels. Parasympathetic nerve cell bodies are located in the brainstem and enter the gut via the vagus nerve or spinal cord [23]. The ascending and proximal two-thirds of the transverse colon receive sympathetic innervation through the superior mesenteric ganglion and parasympathetic innervation through the vagus nerve. In contrast, the distal one-third of the transverse colon, descending colon, sigmoid colon, and upper rectum receive sympathetic innervation via the inferior mesenteric ganglion. The lower rectum receives sympathetic innervation via the inferior hypogastric ganglion, with common parasympathetic input through the pelvic splanchnic nerve [24]. Sympathetic activation leads to the release of noradrenaline (NA), ATP, and neuropeptide Y, which delay intestinal transit and secretion, inhibit smooth muscle cell contraction and secretion, and induce intestinal vasoconstriction [25, 26]. In contrast, parasympathetic nerves exert both excitatory and inhibitory control over gastrointestinal tone and motility, primarily by releasing acetylcholine (Ach) [24].

### Intrinsic innervation

Intrinsic innervation is provided by the enteric nervous system (ENS). The human ENS contains 200–600 million neurons, significantly more than other parts of the peripheral autonomic nervous system [27]. The ENS comprises a vast network of enteric neurons and enteric glial cells, organized in ganglia and interconnected by nerve fiber bundles. Enteric nerve fibers closely interact with the intestinal epithelium and innervate the entire gut wall, controlling gut secretion, reabsorption, and motility through signaling involving both enteric neurons and enteric glial cells [28, 29]. ENS neurons are distributed in thousands of small ganglia, primarily arranged in two major plexuses: the submucosal plexus (SMP) and the myenteric plexus (MP) [30]. The SMP is located in the small and large intestine, composed of an inner plexus at the border of the muscularis mucosae and the submucosa, and an outer plexus adjacent to the circular muscle (CM). The MP extends from the upper esophagus to the internal anal sphincter, situated between the circular and longitudinal muscle (LM) layers [31]. The ENS can autonomously control many digestive functions through the interaction between enteric neurons and glial cells. Gastrointestinal (GI) function is maintained in the absence of vagal or sympathetic connections, but ENS neuropathies can have life-threatening consequences as propulsion of content in the affected region is ineffective [31, 32]. Therefore, the ENS is often referred to as “the brain of the gut”.

Although the ENS plays a significant role in GI function, input from the CNS is also important. There is a bidirectional information flow between the ENS and CNS, and they combine signals to collectively maintain bowel contractility and secretion.

## Clinical relevance of PNI

**Fig. 2:**
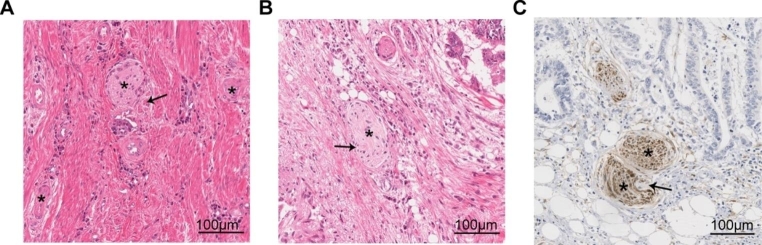
Perineural invasion in human colorectal cancer specimens. (**A**, **B**) Sections are stained with hematoxylin and eosin (H&E). Tumor cells surround and locate within the peripheral nerve sheath; (**C**) The expression of pan-neuronal marker PGP9.5 is detected by immunohistochemical. Asterisks indicate nerve fibers; arrows point to cancer cells invade to nerves sheath

PNI has been recognized as a pathological feature of CRC in numerous studies. It has become a standard component of CRC pathology reporting [33], and the AJCC-UICC 8th edition of TNM staging recognizes PNI as an additional prognostic factor related to the tumor. However, the lack of uniform reporting standards and guidelines for PNI has resulted in the usage of various definitions in different studies [12]. Figure 2 illustrates representative histological images of PNI in human CRC tissues using hematoxylin and eosin (H&E) staining and immunohistochemical staining.

Generally, PNI serves as a prognostic factor associated with tumor metastasis, recurrence, overall survival (OS), and disease-free survival (DFS) in CRC (Table 1). However, some studies have shown conflicting results, suggesting that PNI may not always be an independent predictor of tumor recurrence (overall and early) and patient survival [17, 34]. These discrepancies are likely attributed to the retrospective design of those studies and the high variability in histological assessment. PNI is linked to aggressive features of CRC, including larger tumor size, deeper invasion, lymph node involvement, poor differentiation, and the presence of distant metastases. Previous studies have reported varying incidence of PNI, approximately 10% in stage I-II CRC, up to 30% in stage III, and up to 40% in stage IV disease [18, 35].

Lymph node involvement (LNI) is another pathologic feature associated with adverse disease prognosis and cancer-related mortality in CRC. Some studies support PNI as a predictor of LNI at the time of diagnosis [35, 36]. Additionally, the presence of PNI is associated with tumor lymph node metastasis [37], which may be particularly relevant to large caliber axons often in contact with lymph nodes. Notably, in CRC cases without perineural and lymph infiltration, the 5-year survival rate of patients significantly improves [38, 39]. The strong correlation between PNI and lymph node metastasis warrants further investigating.

The National Comprehensive Cancer Network’s Clinical Practice Guidelines identify PNI as a high-risk factor for CRC recurrence and recommend adjuvant therapy for patients with stage II and PNI-positive CRC [40]. Additionally, adjuvant chemotherapy has been shown to extend 5-year DFS and attenuate the adverse effects of PNI on survival in patients with stage II-III CRC [41–43]. Therefore, PNI status could be used to identify stage II-III CRC patients who would benefit from adjuvant chemotherapy [42].

**Table 1:** The prognostic value of PNI.

Cancer type	Year	Outcome measure associated with PNI	References
CRC	1988	Metastases rate is 72.7% in PNI-positive patients versus 27% in PNI-negative patients (P < 0.01); 3-year OS rate is 29.6% as opposed to 57.7% in those without PNI (P < 0.003).	[44]
	2004	In patients with lymph node-negative CRC, PNI is significant prognostic factors according to the multivariate analysis; the 5-year survival rate is 87% for PNI-negative patients versus 57% for PNI-positive patients (P < 0.006).	[45]
	2015	PNI is associated with poor prognosis in OS (P < 0.01) and DFS (P < 0.01).	[46]
	2020	PNI is an independent high-risk factor of lymph node metastasis.	[36, 47]
Rectal cancer	1990	PNI is a prognostic factor that predicts significantly reduced actuarial survival rates in patients with node-negative tumors.	[48]
	1993, 1998, 1999	The cases with PNI show a higher recurrent rate and worse prognosis compared with those without PNI.	[16, 49, 50]
	2004	27% 5-year cancer-specific survival rate in PNI-positive tumors versus 78% in PNI-negative tumors (P < 0.001).	[51]
	2019	PNI is an independent prognostic factor for 3-years DFS and 3-years OS (P < 0.001).	[52]
	2020	PNI is associated with lower 5-years OS rates in stage I-III patients (P < 0.01).	[53]
	2021	PNI is associated with T and N downstaging after preoperative chemoradiotherapy (P < 0.001); 5-year DFS rate is 59.0% in PNI-positive group, compared to 80.2% in the negative group (P = 0.001).	[54]
Colon cancer	2001	PNI is associated with a high risk of tumor recurrence (P = 0.02).	[17]
	2014	The 5-year DFS rate is greater for PNI-negative patients compared with PNI-positive patients in T3N0 tumors (92.0% versus 76.0%, P = 0.025).	[55]
	2019	Patients with PNI-positive and lymph node-negative have worse OS than patients with PNI-negative and lymph node-positive (p < 0.001); PNI is significantly associated with worse DFS (P = 0.033), worse OS (P < 0.001), and worse DFS (P = 0.048).	[56]
	2019	PNI is an independent factor associated with poor recurrence-free survival (P = 0.003); obstruction is more frequent in PNI-positive cancer (39%) than in PNI-negative cancer (24%, P = 0.0003).	[57]
	2021	PNI is independent prognostic factors for 5-year DFS in T2 and T3 tumors (P = 0.019).	[58]

## Molecular mechanism underlying PNI

**Fig. 3:**
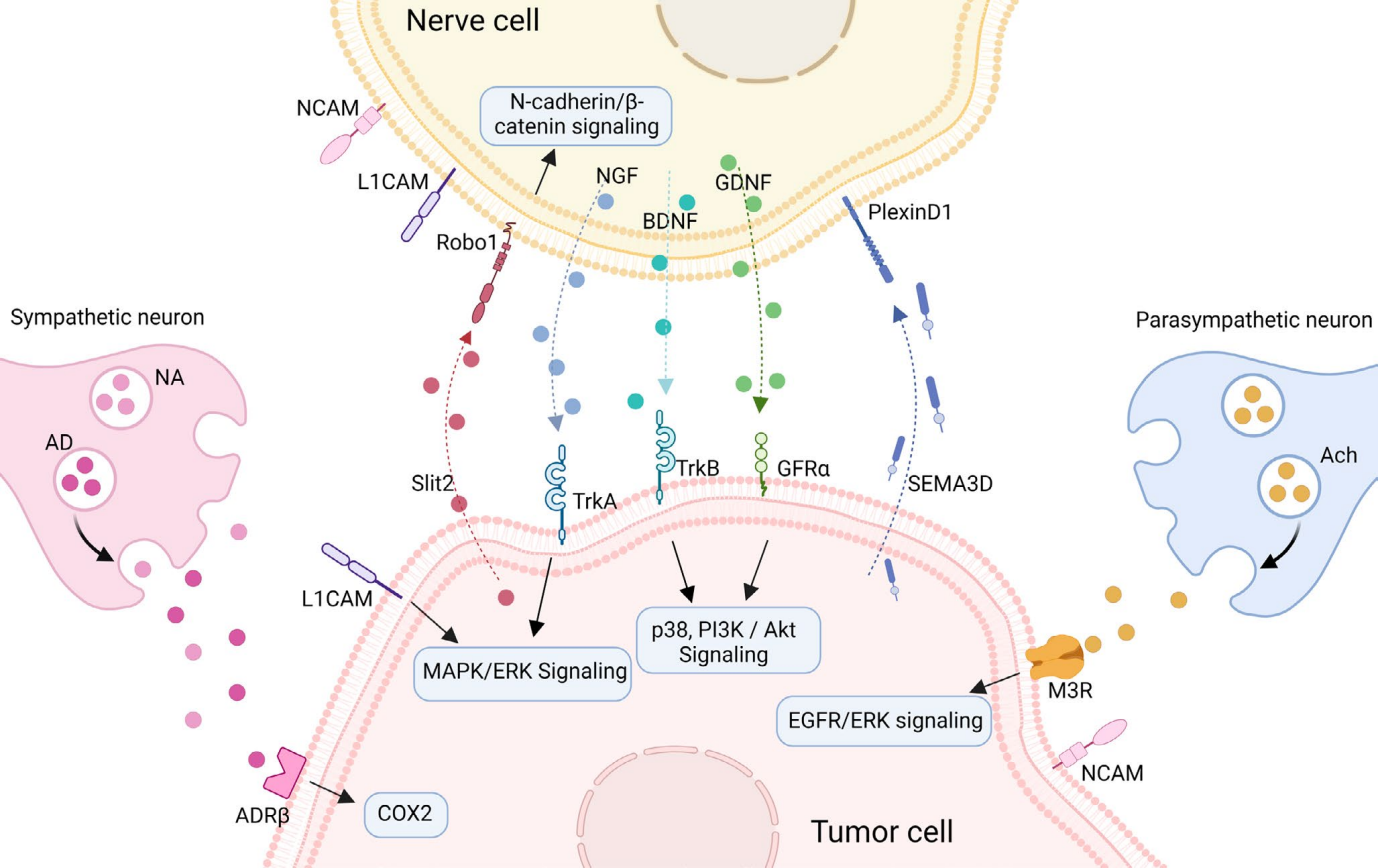
The partial interaction mechanism between CRC cell and neurons. Neural cells could secret neurotrophic factors such as NGF, BDNF, and GDNF, which act on corresponding receptors TrkA, TrkB, and GFRα, thus promoting tumor proliferation and invasion. In turn, tumor cells release axon guidance molecules Slit2 and SEMA3D, which bind to receptors Robo1 and PlexinD1, inducing neurite outgrowth. Transmembrane proteins NCAM and L1CAM facilitate tumor-nerve adhesion, providing a possible route for PNI. Sympathetic and parasympathetic neurotransmitters act on adrenergic receptor and cholinergic receptor respectively, regulating tumor cell proliferation and progression

The pathogenesis of tumors appears to be a deliberate and reciprocal process between cancer cells and the surrounding microenvironment. This process is initiated and driven by molecular signals that promote cancer cell survival, proliferation, and invasion. Additionally, these signals induce neurogenesis and axonogenesis, contributing to the complex interplay between tumor cells and the neural components of the TME. (Fig. 3).

### Neurotrophins

Neurotrophins are a family of proteins that, together with their receptors, promote the survival, growth, and function of nerve cells [12]. This family includes nerve growth factor (NGF), brain-derived neurotropic factor (BDNF), glial cell line-derived neurotrophic factor (GDNF), neurotrophin-3 (NT-3) and neurotrophin-4/5 (NT-4/5) [59]. Neurotrophins are generated by nerve-governed tissues, astrocytes, or tumor cells, exerting direct effect on the interaction between cancer cells and nerves in the TME [60].

NGF has been implicated in PNI in multiple cancers [61]. NGF induces phosphorylation of its high-affinity receptor tropomyosin-related kinase A (TrkA), leading to the activation of the MAPK/ERK signaling pathway, which promotes CRC metastasis [62]. Moreover, the expression of NGF in CRC tissues correlates with TrkA, matrix metalloproteinase 2 (MMP2), and MMP9 [62]. In a chemically induced colon cancer model (Azoxymethane/Dextran sulfate sodium mouse inflammatory CRC model), overexpression of NGF in the colon epithelium resulted in the development of more and larger tumors, suggesting a potential role of NGF in CRC development [63]. Moreover, NGF facilitates the innervation of perivascular nerves, regulating the blood flow in solid tumor neovessels, which may be associated with changes in perivascular nerves within tumors and the adjacent submucosa of CRC [64, 65].

BDNF is widely expressed in the mammalian brain and primarily mediates brain development and synaptic plasticity through its cell surface receptor, tropomyosin-related kinase B (TrkB) [66]. In colon carcinoma, both BDNF and TrkB are upregulated compared to non-tumor tissues, especially in tumors with advanced clinical stages [67, 68]. Additionally, the up-regulation of TrkB in colon cancer cells is correlated with lymphatic vessel metastasis [69]. BDNF and TrkB agonists have also been shown to increase the proliferation of CRC cell lines and exhibit anti-apoptotic activity [67]. Furthermore, BDNF plays a role in enhancing colon cancer cell migration by regulating vascular endothelial growth factor (VEGF)/HO-1 activation through the ERK, p38, and PI3K/Akt signaling pathways [70].

The GDNF family comprises several proteins, including GDNF, neurturin (NRTN), artemin (ARTN), and persephin (PSP), each of which binds to a special GDNF receptor-α (GFRα). These molecules are crucial for maintaining neuron growth and also affect the survival, proliferation, invasion, and metastasis of cancer cells [71, 72]. It has been reported that the GDNF expression in colon adenocarcinoma might contribute to intestinal ganglioneuromatosis [73]. Furthermore, GDNF-induced integrin expression significantly impacts CRC cells’ invasion of the extracellular matrix [74]. Huang et al. reported that GDNF enhances the migration of colon cancer cells by promoting VEGF-VEGFR interaction, primarily regulated by the p38, PI3K/Akt, and HIF-1α signaling pathways [75]. Additionally, NRTN is enriched in CRC cells and is associated with poor patient outcomes. NRTN promotes CRC cells motility and tumor angiogenesis by inducing overexpression of ZEB1/N-cadherin and VEGF-A [76]. Inhibition of NRTN prevents CRC metastasis and angiogenesis in vivo, making it a potential therapeutic target for CRC patients.

### Axon guidance molecules

During tumor development, axonogenesis or neurite outgrowth is enhanced, which is essential for PNI [77]. Axonal growth is a complex process that requires neurotrophic growth factors as well as axonal guidance molecules, such as the netrin, ephrin, semaphorin (SEMA), and slit families [78].

Netrins are laminin-like proteins consisting of four secreted molecules: netrin-1, netrin-3, netrin-4, netrin-5, and two membrane-anchored members: netrin-G1, netrin-G2 [79]. Netrin-1 has been recognized as a potential biomarker for CRC. Zhu et al. demonstrated that serum levels of netrin-1 were significantly higher in CRC patients compared to individuals without tumors or patients with advanced adenoma [80]. Furthermore, the high netrin-1 group had an increased risk of developing CRC when compared to the low netrin-1 group. However, another study indicated that CRC patients exhibit decreased mRNA and serum levels of netrin-1 due to DNA hypermethylation of the *NTN1* gene [81]. It is worth noting that the netrin-1 receptors, deleted in colorectal cancer (DCC) and uncoordinated 5 homolog (UNC5H), are often silenced in CRC through mechanisms such as loss of heterozygosity or epigenetic modifications. The methylation-mediated repression of several *UNC5H* genes holds promise as a diagnostic and prognostic marker in CRC [82, 83].

Ephrin/Eph signaling plays a crucial role in the regulation of cell adhesion, migration, and sorting. Ephrin-A proteins are anchored to the cell surface by a glycosylphosphatidylinositol anchor and interact with class A receptors (EphA), while ephrin-B proteins are transmembrane proteins that bind to class B receptors (EphB) [84]. Specifically, ephrin-A1 expression serves as a valuable marker for predicting a higher risk of recurrence and cancer-related mortality in patients who have undergone curative resection for CRC [85]. In vitro assays have demonstrated that decreased ephrin-A1 expression is associated with reduced proliferative activity, as well as decreased invasion and migration of CRC cell lines [85]. Furthermore, research has shown higher expression of ephrin-B2 in colon carcinoma compared to adjacent normal tissues [86]. Additionally, ephrin-B1 and ephrin-B2 are preferentially incorporated into exosomes derived from CRC [87]. These findings suggest that ephrin-B1 and ephrin-B2 could potentially serve as diagnostic biomarkers. Interestingly, the overexpression of ephrin-B2 in CRC has been found to be associated with increased tumor angiogenesis, unexpectedly resulting in reduced tumor growth due to the structural abnormalities of the new vessels [88].

SEMA proteins serve as cues for axons to navigate through their environment, with some acting as attractants and others as repellants [89]. SEMA3C is implicated in the development of the ENS. A loss-of-function mutation in *SEMA3C* leads to Hirschsprung’s disease, a congenital disease in which the ENS fails to form in parts of the intestine [90]. In Crohn’s disease, the increased SEMA3C expression in intestinal crypts is associated with a reduction in mucosal sympathetic nerve fibers [91]. SEMA3C is also involved in various oncogenic processes in colon, gastric, lung, liver, breast, and pancreatic cancers [92]. SEMA3D also plays a role in the formation of neuronal networks. Reducing SEMA3D or its receptor plexin D1 expression inhibits the invasion of tumor cells towards the nerves and decreases the nerve density in tumor tissues [93]. Additionally, SEMA4D and its receptor plexin B1 are highly expressed in tumor cells and nerves, respectively, promoting PNI in colon cancer [94].

Slits (Slit1, Slit2, Slit3) are secreted glycoproteins that bind to the membrane-bound neuronal guidance receptors Roundabout (Robo) family. The Slit-Robo pathway plays important roles in neuronal guidance and tumorigenesis [95]. Tomasini et al. discovered that Slit2 secreted by cancer-associated fibroblasts could increase dorsal root ganglion (DRG) neuron neurite outgrowth and Schwann cell proliferation/migration by regulating N-cadherin/β-catenin signaling [96]. Several studies have shown that Slit2 is downregulated in CRC tissues compared to adjacent tissues and can inhibit CRC cell migration in Robo-dependent manners [97, 98]. However, Yao et al. found that the serum levels of Slit2 were significantly increased in CRC patients compared to healthy controls. Blocking the binding of Slit2 to Robo1 could inhibit CRC cell migration and metastasis through the TGF-β/Smads signaling pathway [99]. Therefore, further exploration is needed to understand the functions of Slits in CRC.

### Adhesion proteins

Cell adhesion molecules are a group of transmembrane proteins that mediate cell-cell and cell-extracellular matrix interactions. The neural cell adhesion molecule (NCAM), belonging to the immunoglobulin superfamily of adhesion molecules, is expressed on the surface of neurons and Schwann cells [100]. During nervous system maturation, NCAM plays an important role in facilitating neuronal migration, axon/dendrite outgrowth, and synaptogenesis [100]. NCAM is also implicated in tumor growth and metastasis and is associated with PNI in various types of cancers [101–103]. A recent study provided evidence that NCAM expression by Schwann cells serves as a guiding mechanism for cancer cells to migrate towards nerves, thereby promoting cancer cell invasion and dispersion, ultimately facilitating PNI [104]. Previously, NCAM was believed to act as a tumor suppressor in CRC; tumors lacking NCAM expression were associated with aggressive clinical behaviors [105]. However, subsequent studies have demonstrated that NCAM expression is elevated in human CRC tissues and cell lines, and its expression is positively correlated with the presence and number of lymph node metastases [106, 107]. Moreover, the *NCAM* gene has been identified as a target of β-catenin, and the induction of *NCAM* transcription is thought to play a role in colon cancer tumorigenesis, probably by promoting cell growth and motility [107].

The L1 family of cell adhesion molecule (L1CAM) shares important structural and functional features with NCAM and is also involved in signaling transductions related to neuron migration and neurite outgrowth [108]. L1CAM upregulates cell-matrix adhesion by initiating the MAPK ERK1/2 signaling downstream cascade, which triggers the transcription of integrins, thereby enhancing cell motility [109]. In CRC, L1CAM has been proven to be a potential marker for tumor invasiveness, metastasis, lymph node metastasis, and worse patient outcomes [110–112]. Moreover, L1CAM mRNA expression is significantly higher at the invasive front compared to the center of the tumor, indicating a supportive role of L1CAM in CRC dissemination [113]. L1CAM can also mediate tumor-nerve adhesion, allowing CRC cells to migrate along enteric neurons in vitro and potentially contributing to PNI [114].

### Neurotransmitters

During tumor development, cancer cells and peripheral nerves release numerous neurotransmitters directly into the TME, which can activate corresponding receptors and influence various cellular signaling pathways, promoting cancer cell proliferation and progression. Neurotransmitters are mainly classified into four types based on their chemical composition: choline (Ach), catecholamines (adrenaline, NA, and dopamine), amino acids (excitatory transmitter such as glutamic acid and aspartic acid; inhibitory transmitters such as γ-aminobutyric acid and glycine), and neuropeptides (vasoactive intestinal peptide) [115]. These neurotransmitters play critical roles in maintaining various physiological functions and stress responses.

Adrenergic signaling is reported to be upregulated in CRC. Pathology reports have shown that β adrenergic receptors (ADRβ) are more abundant in the tumor site, which is usually correlated with worse prognosis [116]. Adrenergic transmitters can promote the proliferation of human colon cancer cell line HT-29 by inducing the expression of cyclooxygenase 2 (COX-2), VEGF, prostaglandin E2, and MMP-9. This effect can be rescued by ADRβ antagonists or COX-2 inhibitors [117]. Furthermore, NA has been shown to stimulate the proliferation and dissemination of colon cancer cells by inducing phosphorylation of cAMP response element-binding protein 1 (CREB1), thereby activating the CREB1/miRNA-373 axis [118]. Additionally, adrenaline (AD) can increase the expression of COX-2 and interleukin (IL)-10 in macrophages, suppressing CD8 + T lymphocytes proliferation and interferon-γ production, thus facilitating immune escape in colon cancer [119].

γ-aminobutyric acid (GABA) is a neuromodulator that helps restore homeostasis. It inhibits pro-inflammatory cytokines, stimulates anti-inflammatory cytokines, decreases intestinal permeability, and promotes neuronal survival [120]. The GABA shunt involves the enzymatic conversion of glutamate to GABA by glutamate decarboxylase 1 (GAD1) and GAD2, followed by the catabolism of GABA by GABA-transaminase (ABAT). Notably, GAD1 expression is upregulated in CRC cell lines and tumor tissues, while ABAT expression is downregulated, suggesting the accumulation of GABA in CRC may be attributed to elevated GAD1 and a lack of ABAT [121]. In a mouse model of colon cancer, GABA can directly bind to the GABA type A receptor (GABA_A_R) on CD8 + T lymphocytes, reducing anti-tumor immunity and facilitating tumor growth [122]. Additionally, Huang et al. discovered that GABA activates the GABA type B receptor (GABA_B_R), leading to the enhancement of β-catenin signaling, promoting CRC cell proliferation, and inhibiting the intratumoral infiltration of CD8 + T lymphocytes, resulting in immunosuppression [121]. Furthermore, lower GABA_B_R1 expression in tumor tissues impairs CRC cell migration and invasion by regulating epithelial-mesenchymal transition, while higher GABA_B_R1 expression is associated with longer survival in CRC patients [123]. However, other studies show that GABA acting at the GABA_B_R can inhibit colon cancer cell migration in vitro, enhance the anti-tumor efficacy of oxaliplatin, and reduce metastasis of CRC in mice [124, 125].

A vast number of enteric neurons are cholinergic and have excitatory or inhibitory control over gastrointestinal motility by releasing Ach [24]. Parasympathetic innervation via Ach and its receptors is observed in later stages of CRC and is associated with a poor prognosis, suggesting that cholinergic parasympathetic nerves could signal to CRC tissue and exacerbate the disease [126]. Muscarinic Ach receptors are the predominant Ach receptors distributed in the gut and can induce the growth of colon cancer by activating the EGFR/ERK signaling pathway, with muscarinic Ach receptors 3 (M3R) significantly overexpressed in CRC lesions [127]. The activation of nicotinic Ach receptors leads to increased cell proliferation and decreased apoptosis in human colon cell lines [128, 129].In gastric cancer, Ach released by cholinergic nerves and Tuft cells contributes to tumorigenesis, stimulating the production of NGF from epithelial cells, which further increases nerve density and Ach release in turn [63]. Notably, Ach can also be released by colon cancer cells in an autocrine manner [130], making it more complicated to observe neuron-cancer crosstalk.

The first neurotransmitter associated with CRC is vasoactive intestinal peptide (VIP) [131]. VIP is widely expressed in the central and peripheral nervous system, as well as in the gastrointestinal tract. In peripheral nerve crush or transection models, an increase in VIP immunoreactivity and mRNA expression has been observed, indicating the involvement of VIP in the process of nerve repair [132, 133]. In the gastrointestinal tract, VIP primarily acts as an intestinal relaxant and reduces intestinal permeability to counterbalance the effects of permeability-increasing factors such as Ach [134]. The role of VIP in the intestinal epithelium is controversial. On the one hand, the addition of VIP to cultures of the CRC cell lines stimulates cell proliferation via the activation of the MAPK pathway in a time- and concentration-dependent manner [135]. In an azoxymethane-induced mice CRC model, the administration of VIP before and during azoxymethane treatment leads to a significant increase in the incidence of colonic tumors [136]. On the other hand, contradictory evidence indicates that VIP attenuates the motility and invasiveness potential of colon cancer cells [137], and inhibits liver metastasis partly due to the prevention of tumor angiogenesis [138]. The mechanisms underlying these different effects of VIP are yet to be determined.

## Cells in TME related to PNI

**Fig. 4:**
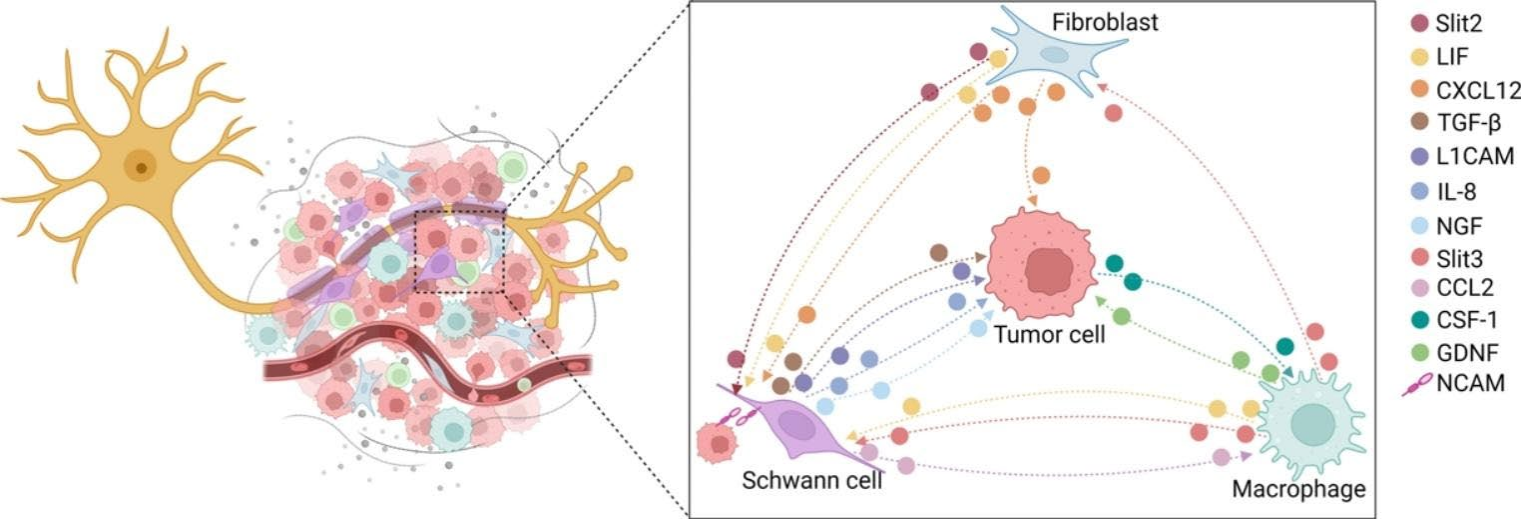
Cellular crosstalk in the TME. Schwann cells regulate tumor cell proliferation, invasion, and PNI through membrane protein (NCAM) and secretory proteins (TGF-β, LICAM, IL-8, NGF). Tumor cells and Schwann cells secrete CSF-1 or CCL2 to recruit macrophages to the site of PNI, which aggravates nerve injury and tumor invasion. In turn, macrophages release GDNF, promoting cancer migration. Macrophages also secret Slit3, which guides Schwann cells and fibroblasts in forming a peripheral nerve bridge during nerve injury. Both macrophages and fibroblasts can release LIF, which promotes Schwann cell migration and neural plasticity. Fibroblasts also stimulate Schwann cell proliferation and neural remodeling via secreting Slit2. Additionally, the combination of CXCL12 and CXCR4 induces Schwann cell migration and infiltration

Emerging evidence highlights the critical role of TME in tumor progression [139]. The involvement of the nervous system within the TME has also gained attention [140–144]. Most cells in the TME express receptors for neurotransmitters such as NA and Ach, indicating that the crosstalk between different cells and neurons can potentially affect the pathogenesis of tumor. In this review, we will focus on the major cell types that support nerve-cancer interactions within and adjacent to the peripheral nerve sheath (Fig. 4).

### Schwann cells

Schwann cells, originating from neural crest cells, are the neurogliocytes of the periphery nervous system [145]. They are essential components of the nerve microenvironment, playing crucial roles in nurturing neurons during development, supporting neuronal repair, and regeneration processes [60, 146]. There is evidence that Schwann cells can migrate towards colon cancer even before any neural invasion, suggesting their involvement in initiating PNI [147]. During PNI, Schwann cells interact with tumor cells directly through plasma membrane proteins such as NCAM and indirectly via secretory proteins such as L1CAM and transforming growth factor-beta (TGF-β), thereby augmenting the aggressive capacity of tumor cells and promoting PNI in vitro and in vivo [104, 148, 149]. After contacting cancer cells, Schwann cells can intercalate between cancer cells, stimulating tumor protrusion and dispersion. These processes promote cancer cells migration and invasion along nerves [104]. Schwann cells can also assemble into “tumor-activated Schwann cell tracks”, envelop cancer cells and enhance their motility along the pathways of tissue innervation [150]. Additionally, Schwann cells secrete C-C motif chemokine ligand (CCL) 2, which recruits macrophages to the site of PNI, degrades collagen IV of the nerve perineurium, and ultimately aggravates nerve injury and tumor invasion [151]. Notably, recent studies have demonstrated reciprocal communication between Schwann cells and CRC cells. IL-8 and NGF from Schwann cell-conditioned medium could promote CRC proliferation, invasion and metastasis, and may serve as potential therapeutic targets for the treatment of CRC [152, 153].

### Immune cells

The interaction among cancer cells, nerves, and immune cells is essential for tumor progression. During tumorigenesis, cancer cells secrete colony-stimulating factor 1 (CSF-1) to recruit macrophages, which, in turn, release GDNF, promoting cancer migration as well as nerve invasion [154]. It has been reported that tumor-associated macrophages (TAMs), a pro-tumor M2 subtype of macrophage, express more GDNF than resting macrophages [154]. Muscularis macrophages have been shown to interact directly with enteric neurons and enteric glial cells, regulating inflammation and peristaltic activity of the colon [155, 156]. Macrophages can secret Slit3, which guides Schwann cells and fibroblasts in forming an appropriate peripheral nerve bridge, ensuring axon targeting to the distal nerve stump following injury [157]. Besides, both macrophages and fibroblasts secrete the leukemia inhibitory factor (LIF), which promotes Schwann cell migration and neural plasticity, leading to enhanced neurite outgrowth [158].

Lymphocytes exert both pro- and anti-tumor effects during the dynamic modulation of the TME. At the intestinal mucosal barrier, lymphocytes have been observed to co-localize with neurons, glial cells, and neuroendocrine cells, and the crosstalk between lymphocytes and nerves maintains mucosal homeostasis [159]. Famulski et al. demonstrated a negative correlation between PNI and the infiltration of stromal tumor-infiltrating lymphocytes (TILs) in CRC, while no correlation was found with intraepithelial TIL infiltration [160]. Similarly, Huh et al. observed a correlation between a low proportion of TILs and the occurrence of PNI in CRC [161]. Moreover, CRC tumors without early metastatic invasion exhibit increased numbers of CD8 + T lymphocytes, along with elevated expression of markers associated with T lymphocyte migration, activation, and differentiation [20]. Mechanistically, the input of sympathetic nervous system into the TME inhibits cancer cell production of type I interferon and the recruitment of M1 macrophages, natural killer cells, and CD8 + T lymphocytes, thereby compromising tumor immune surveillance and restraining anti-tumor inflammation [162]. Conversely, the parasympathetic nervous system, specifically the vagus nerve, can modulate the immune microenvironment through cholinergic signaling, promoting tumor growth by impairing CD8 + T lymphocytes infiltration and Th1 differentiation, partly through HDAC-mediated inhibition of CCL5 expression [163].

### Fibroblasts

Cancer-associated fibroblasts (CAFs), the predominant stromal cells in CRC, can release growth factors, cytokines, pro-angiogenic factors, and extracellular matrix proteins, which contribute to carcinogenesis, angiogenesis, and PNI [164–166]. Importantly, fibroblasts are the main cellular constituents of the perineurium [167]. Alterations in the perineurium composition may increase its permeability and compromise the protective barrier against tumor cell invasion. In CRC, CAFs release stromal cell-derived factor 1, also known as C-X-C motif chemokine (CXCL) 12, which has been shown to accelerate CRC metastasis and cisplatin resistance [168, 169]. The CXCL12 and its receptor C-X-C motif chemokine receptor (CXCR) 4 axis can induce tumor infiltration by Schwann cells during early carcinogenesis and enhance Schwann cells migration via the p38/MAPK signaling pathway [170, 171]. Activation of CXCL12/CXCR4 axis significantly promotes cancer cell invasion and facilitates the outgrowth of DRG, eventually leading to PNI [172]. CAFs also secrete Slit2, which stimulates Schwann cell proliferation and neural remodeling, resulting in increased nerve density [96]. The expression of fibroblast specific protein-1 (FSP-1) in CAFs within the intra-tumoral stroma is associated with PNI, lymphatic invasion, and tumor stage of CRC [173]. Another widely used marker for CAFs in various tumor types is fibroblast activation protein alpha (FAPα), and its expression is correlated with PNI and poor prognosis [174]. In a study by Tassone et al., fibroblasts within the PNI microenvironment were found to express MMP-2, whereas fibroblasts in nerves without PNI did not, suggesting that MMP-2 expression by fibroblasts may be a potential mechanism promoting PNI [175].

## Models to study PNI

Our current understanding of PNI pathogenesis has been constrained by the absence of effective models capable of capturing the intricate interactions between nerves, tumor cells, and the stroma. In our previous review, we provided an overview of commonly used in vitro and in vivo models employed in PNI research [176]. In this review, we will specifically highlight models utilized to investigate PNI in CRC and explore potential novel models for studying enteric nerves.

### In Vitro models

Duchalais et al. initially cocultured tumor epithelial cells from human primary colon adenocarcinomas with human ENS plexus explants in a Transwell chamber coated with Matrigel to mimic tumor cells invasion into the neural sheath [114]. In this study, confocal and atomic force microscopy, as well as video microscopy, were employed to assess colon cancer cell adhesion and migration on the ENS. More recently, a colon-nerve organoid model was utilized to investigate the reciprocal signaling between neurons and cancer cells [177]. This model involved using L6 dorsal and ventral roots with the spinal cord removed from the spinal column. Through this model, the authors demonstrated that extrinsic parasympathetic pathways influence myenteric neuron activity and mediate smooth muscle contractions in the colon. This method can be adapted for studying sensory neuron hypersensitivity by recording neuronal activity electrophysiologically in the presence of cancer cells.

Tissue clearing followed by three-dimensional (3D) imaging is a technique that enables the simultaneous analysis of multiple cells within an organ. Masaki Mori et al. were the first to characterize the 3D structure of tumor invasion around nerve tissue in CRC using this method [178]. By staining CRC tissue with cytokeratin AE1/AE3 and anti-S100 protein antibody, virtual slides were scanned to reconstruct the tissue, effectively demonstrating the morphological features of intramural PNI in CRC [178].

In vitro experimental models allow investigators to control the experimental components and conditions of the cellular microenvironment, and capture specific aspects of the disease process. However, these models have limitations, particularly in replicating the neural microenvironment and culturing peripheral nerves.

### In vivo models

The ideal in vivo models should recapitulate the neuropathic changes that occur in human tumors, such as neural hypertrophy and neural remodeling. Genetically engineered mouse models (GEMMs) are valuable tools for elucidating the molecular mechanisms underlying tumor initiation and development and have shown great promise in investigating PNI [179, 180]. GEMMs allow researchers to observe the neurogenesis, neural remodeling, and tumor-neural interactions at different stages of tumor progression. This can be achieved through the following methods: (1) Immunohistochemistry and immunofluorescence staining can be performed on tissues to label nerves and tumor cells. Additionally, tissue clearing followed by 3D imaging can be employed for neural reconstruction. (2) The expression levels of neurotrophic factors, neurotransmitters, chemokines, and other molecules related to neural function can be measured in tumor tissues and/or blood samples. Correlation analysis can be performed to examine their association with tumors and neural features. (3) The expression levels of RNAs and proteins in tumors can be examined to assess their correlation with PNI and other neural characteristics. (4) Researchers can test the effectiveness of drugs targeting specific molecular or pathways involved in PNI.

The most widely utilized GEMM of intestinal cancer is the *Apc*^*Min/+*^ model, which harbors a dominant nonsense mutation in one *Apc* allele, resulting in the development of multiple intestinal adenomas, primarily in the small intestine, with few occurring in the colorectum. Vasiliou et al. crossed *Apc*^*flox*^ mice with *Cdx2*^*ERT2 − Cre*^ mice to generate deficient *Apc* specifically in colon epithelial cells following tamoxifen treatment [181]. Kevin G et al. generated *Apc*^*Min/+*^; *Kras*^*LSL−G12D/+*^; *Villin*^*ERT2−Cre*^ compound mutant mice, carrying a Cre-dependent activated *Kras*^*LSL−G12D*^ on the *Apc*^*Min/+*^ background, crossed with mice carrying the *Villin*^*ERT2−Cre*^ transgene, which expresses Cre recombinase throughout the intestine, and confirmed an enhancement of tumor development in the colon [182]. Similarly, an *Apc*^*flox*^; *Kras*^*LSL−G12D/+*^; *TP53*^*KO/KO*^; *Villin*^*ERT2−Cre*^ compound mutant mice model was used to study cancer stem cells in CRC [183]. Although GEMMs allow us analyze the complex interactions between nerves, tumor cells, and other cells in the TME, the incidence of PNI in them has not been reported.

Other in vivo models include the orthotopic model and heterotopic model. The orthotopic model has been used in the study of pancreatic and head and neck cancer, and the incidences of PNI have been recorded [184, 185]. The widely used heterotopic model is mouse sciatic nerve model, which can recapitulate cancer cell migration through invaded nerves [151, 186]. However, neither of these models is suitable for studying the interactions between cancer cells and nerves at pre-neoplastic stages. Furthermore, the sciatic nerve is a somatic nerve, which is different from the natural innervation of the actual end-organ. Thus, it is urgent to develop other models that can recapitulate the development of PNI.

## Therapeutic potential

The strong clinical impact of PNI makes it a promising therapeutic target. While there have been limited active clinical trials on CRC patients so far, animal models have been used to investigate the main molecules involved in PNI. Under stress conditions, human CRC cells synthesize BDNF, which stimulates cell proliferation and exerts an anti-apoptotic effect. This effect can be suppressed by K252a, a pharmacologic inhibitor of Trk receptors [187]. In murine xenograft models, targeting BDNF/TrkB signaling with K252a results in reduced metabolic activity and enhanced apoptosis of CRC cells [188]. In the TrkA-expressing CRC cell line KM12, the selective TrkA inhibitor NMS-P626 effectively inhibits TrkA phosphorylation and downstream signaling, demonstrating significant antitumor activity in mice with xenograft tumors [189]. Additionally, inhibition of GDNF expression by miR-196a-5p mimics has been shown to reduce the migration of CRC cells in vitro [190]. In a study by Duchalais et al., blocking L1CAM and N-cadherin with antibodies resulted in decreased migration of human primary colon adenocarcinomas epithelial cells along ENS structures [114]. The CCL2 and C-C motif chemokine receptor (CCR) 2 axis has also been implicated in PNI through Akt and MAPK signaling pathways. Treatment with anti-CCL2 antibodies has been found to inhibit CRC angiogenesis and growth by blocking p38/MAPK signaling [191]. Although MMPs are considered potential therapeutic targets in PNI, several clinical trials have failed to demonstrate significant effects on tumor development using broad-spectrum MMP inhibitors [192].

Targeting tumor innervation has also been proposed as a therapeutic option for several cancers, including breast cancer [142], prostate cancer [8], and gastric cancer [143]. In CRC, denervation of the myenteric plexus through benzalkonium chloride (BAC) treatment reduces the number of both preneoplastic and neoplastic lesions, suggesting that colonic denervation could attenuate carcinogenesis in the early stages [193, 194]. Targeting M3R via selective or non-selective antagonists could repress colon cancer cell proliferation, whereas acetylcholinesterase inhibitors are capable of stimulating tumor growth [130]. Similarly, deficiency of *CHRM3*, the coding gene of M3R, reduces the epithelial proliferation and tumor size in murine colon cancer models [195]. ADRβ is described as a potential therapeutic target in multiple cancers as it can promote tumor progression when activated by sympathetic signaling [196]. Selective ADRβ antagonists suppress CRC cell proliferation and viability both in vivo and in vitro, probably through the EGFR-Akt/ERK1/2 signaling pathway [197]. Wang et al. found that trefoil factor 2 (TFF2) is essential for the anti-inflammatory neural arc involving the vagus nerve and memory T cells. Deletion of *Tff2* or splenic denervation disrupts this arc, leading to pro-carcinogenic inflammation in CRC [198]. These results indicate the participation of cholinergic and adrenergic signaling in CRC development, suggesting the therapeutic potential of denervation in cancer treatment.

## Discussion and perspectives

Despite the dense innervation of the colon and rectum, the prevalence of PNI in CRC remains low, and the reasons for this phenomenon are not yet fully understood. One possible explanation is that the natural organ-innervation of the tumor origin may lack significant biologic affinity between cancer cells and neurons. The expression of neurotrophins, chemokines, and their receptors in colonic nerves and tumor cells is much lower in CRC compared to PDAC [199]. Additionally, the absence of remarkable neuroplasticity in CRC may contribute to the low occurrence of PNI. Furthermore, PNI is often underreported due to the lack of standardized reporting standards. The incidence of PNI in rectal cancer is higher than in colon cancer, which can be attributed to the presence of numerous autonomic nerve plexuses surrounding the rectum [18]. The rectum is localized extra-peritoneally and possesses its own rectal plexus, while several parts of the colon are intraperitoneal and lack an external plexus, resulting in differences in innervation density [200]. Moreover, more extensive examination of the mesorectal fat in rectal cancer to investigate circumferential resection margin involvement may increase the detection rate of PNI.

Despite the relatively low prevalence of PNI in CRC, its presence exerts a significant influence on the prognosis of the disease, comparable to well-established prognostic factors such as tumor invasion depth and lymphatic invasion. The significance of PNI in CRC lies in its capacity to facilitate tumor invasion along nerves, leading to the dissemination of cancer cells to nearby tissues and distant organs. By allowing tumor cells to interact directly with nerves, PNI creates a favorable microenvironment that supports tumor growth and survival. Furthermore, PNI can contribute to cancer-related pain and serve as a valuable pathological marker for early metastasis, offering crucial prognostic information.

Overall, PNI involves complex communication between nerves, cancer cells and other components of the neoplastic microenvironment. These reciprocal interactions contribute to tumor growth, migration, dissemination, and metastasis. Understanding the underlying pathological mechanisms of PNI and identifying specific molecular targets may have a potential impact on CRC treatment.

## Data Availability

Not applicable.

## List of Abbreviations

Ach: Acetylcholine
AD: Adrenaline
ADRβ: β adrenergic receptors
BDNF: Brain-derived neurotropic factor
CCL: C-C motif chemokine ligand
CM: Circular muscle
CNS: Central nervous system
COX-2: Cyclooxygenase 2
CRC: Colorectal cancer
CXCL: C-X-C motif chemokine
CXCR: C-X-C motif chemokine receptor
DFS: Disease free survival
DRG: Dorsal root ganglion
ENS: Enteric nervous system
GABA: γ-aminobutyric acid
GABA_A_R: GABA type A receptor
GABA_B_R: GABA type B receptor
GDNF: Glial cell line-derived neurotrophic factor
GEMM: Genetically engineered mouse models
HNSCC: Head and neck squamous cell carcinoma
L1CAM: L1 family of cell adhesion molecule
LM: Longitudinal muscle
LNI: Lymph node involvement
M3R: Muscarinic Ach receptor 3
MMP: Matrix metalloproteinase
MP: Myenteric plexus
NA: Noradrenaline
NCAM: Neural cell adhesion molecule
NGF: Nerve growth factor
OS: Overall survival
PDAC: Pancreatic ductal adenocarcinoma
PNI: Perineural invasion
SEMA: Semaphorin
SMP: Submucosal plexus
TIL: Tumor-infiltrating lymphocyte
TME: Tumor microenvironment
TrkA: Tropomyosin-related kinase A
TrkB: Tropomyosin-related kinase B
VEGF: Vascular endothelial growth factor
VIP: Vasoactive intestinal peptide
